# Risk factors for neck pain among forklift truck operators: a retrospective cohort study

**DOI:** 10.1186/s12891-018-1956-3

**Published:** 2018-02-09

**Authors:** U. Flodin, B. Rolander, H. Löfgren, B. Krapi, F. Nyqvist, C. Wåhlin

**Affiliations:** 1 Occupational and Environmental Medicine, and Department of Clinical and Experimental Medicine, Linköping University, Östergötland, 581 85 Linköping, Sweden; 2 Department of Behavioral Science and Social Work, School of Health Sciences, Jönköping University, Jönköping, Sweden; 3 Futurum, Academy for Health and Care, Region Jönköping County, Jönköping, Sweden; 4 Neuro-Orthopedic Center, Ryhov Hospital, Jönköping, Sweden; 5 Unit of Intervention and Implementation Research, Institute for Environmental Medicine, Karolinska Institutet, Stockholm, Sweden

**Keywords:** Forklift operators, Neck pain, Ergonomics, Work postures, Retrospective cohort study, Occupational medicine

## Abstract

**Background:**

No previous research has been performed into neck pain among forklift operators. This is a common complaint among these workers**,** who number around 150,000 in Sweden and six million in Europe. The aim of the study was to examine long-term exposure to unnatural neck positions among forklift operators as a risk factor for neck pain.

**Methods:**

A retrospective cohort study was conducted of all eligible employees at a high-level warehouse. Forklift operators and office workers answered an 18-page questionnaire comprising questions about joint pain, work tasks, work postures and year of start for all items. By using person years in the exposed and less-exposed groups before start of neck pain we were able to calculate Incident Rate ratios for various exposures.

**Results:**

Forty nine percent of the forklift operators reported having experienced neck pain compared to 30 % of office workers. Being a forklift operator was associated with an increased risk of neck pain (OR = 5.1, 95% CI 1.4–18.2). Holding the head in an unnatural position resulted in significantly increased risks for neck pain, irrespective of type of position. The risks for neck pain remained after taking other ergonomic exposures and psychosocial aspects into consideration.

**Conclusions:**

This is the first published study showing that forklift operators have an increased risk of neck pain. The results are therefore of significance for improving work schedules, the adjustment of work tasks for these workers and the design of the vehicles.

## Background

Risk factors for persistent neck pain in workers are described in a great number of papers. A Swedish review by the Swedish council on health technology assessment was published in 2012 [1]. It focused on the impact of work on the incidence of symptoms and disorders in the neck and shoulders and upper extremities. The review concluded that there is a lack of high quality research to support the association between long-term pronounced flexion, extension or rotation of the head and the development of neck and shoulder pain. This review only scrutinized cohort studies and randomized controlled trials. The cohort studies included in the review covered the general population of Denmark [2–4]; a number of industrial and service sectors [5]; office workers [6]; female nurses [7] and industrial forestry workers [8]. There are at least eight epidemiological studies of forklift operators. However, seven of them do not focus on neck pain [9] and only one addresses neck pain [10]. That study does not, however, evaluate the specific role of neck positions and does not distinguish forklift operators from drivers of earth-moving machines and buses in public transport.

The work of forklift truck operators in large, high-level warehouses involves extension of the neck when loading and unloading the forklift on shelves at high levels – up to 12 levels above the operators. Throughout the workday they repeatedly rotate their heads when reversing the forklift some hundred metres at a time. They also lean to the side to get a better view when loading and unloading their vehicle. Thus, these forklift truck operators are exposed to a variety of non-neutral head positions in their daily work.

Our clinic was given the opportunity to perform a retrospective cohort study on a large group of forklift operators at a large warehouse in southern Sweden. A literature review we performed found no other studies of forklift operators with a specific focus on neck pain. The present study gives us a greater understanding of the association between non-neutral head positions at work and neck pain among forklift operators. The aim of the study was to examine long-term exposure to unnatural neck positions among forklift operators as a risk factor for neck pain.

## Methods

### Study population

This is a retrospective cohort study. All permanently employed staff at a warehouse, which included forklift operators and administrative personnel, were invited to participate. The study was supported by the union and the human resource managers. Four hundred and twenty nine questionnaires were posted to all workers at their home addresses in May 2014. After three postal reminders, 194 subjects (45%) had replied to the questionnaire. The study was approved by the Regional Ethical Review Board at the University of Linköping, Sweden (2014 02 26 Dnr. 2013/418–31).

### Exposure and risk factors

The workplace was first visited by two experienced ergonomists who documented, photographed and filmed the workplace. They were particularly interested in significantly extended, rotated and lateral flexed neck movements among forklift operators. Some of these were interviewed about what they perceived as awkward postures. This information formed the basis for the questions in the questionnaire. Three types of forklift truck are used at the warehouse: the low-lift order picker, the reach decker and the counterbalanced tilting mast. The low-lifting order picker is operated in a standing position; in the other two types of forklift truck the operator is seated. Most of the workers switch between the three. All of them require the operator to hold his/her head in unnatural positions. When reversing the truck the operator rotates the head at least 45 degrees; when loading the fork at high levels he/she holds the head a lateral flexed position, in combination with rotation and extension (backward bending).

### The questionnaire

Exposure information for the employees was collected by means of an 18-page questionnaire containing 41 questions evaluating workplace conditions; demands and health, including job title; start and end of employment; type of forklift truck; work tasks and position of the neck while working. They were also asked about whether they had ever had neck pain; experience of neck pain in the previous seven days; onset and end of neck pain; shoulder pain and a work ability single-item question. Questions about pain and work ability have been used in previous studies [11, 12]. Only self-reported measures were used to classify workers with neck pain and/or shoulder pain for the main outcome. For this study, the definition of having neck pain is based on the question “Do you have or have you had pain, ache or discomfort in your neck?” with a yes/no answer. If yes, the informant gave the year of start and end of symptoms. The definition of having shoulder pain is based on the question “Do you have or have you had pain, ache or discomfort in your shoulders?” with a yes/no answer. If yes, the informant gave the year of start and end of symptoms. Of particular interest were neck positions used in daily work, including neck extension, rotation and lateral flexion and the years of the start and end of exposure to these unnatural positions. General self-reported health was obtained by the question: “In general, would you say your health is…?”, with five anchor points ranging between excellent and poor [13]. The worker’s work satisfaction was measured on a score graded from 0 to 10, where a higher score indicates greater satisfaction. Work-related stress in the previous week was measured on a score graded from 0 to 10, where a higher score indicates higher stress level. Freedom of work was asked by the question: “Do you have the freedom to plan how you do your work?”, using a 4-item scale ranging from “strongly disagree” to “strongly agree”. Co-worker support was also measured by a 4-item scale. The Borg CR10 scale was used to evaluate perceived physically strenuous work [14]. All data were collected in the second quarter of 2014 from the 194 subjects who answered the questionnaire.

### Statistics

Table 1 shows the univariate analyses which were performed on the two cohorts to compare demographics, health and lifestyle, work tasks and psychosocial factors between exposed (forklift operators) and less exposed (office workers). T-tests for prevalence were used for the continuous variables and cross tabulated chi-square tests for the categorical variables. Frequencies were presented in counted numbers with percentages for categorical variables and standard deviation (SD) for continuous variables. Analyses were performed using SPSS, version 23 (IBM SPSS Inc., Armonk, NY). A probability value of 0.05 (5%) was considered statistically significant. A univariate logistic regression analysis was performed (Table 2) using affirmed neck pain as the dependent variable and various potential risk factors as independent variables, in order to calculate odds ratios for neck pain during current employment (OR). Since neck and shoulder pain commonly overlap, a third combined outcome variable for neck and shoulder pain was included in this analysis. To exclude neck pain with no possible connection to the studied exposure, 16 subjects whose pain onset occurred before present employment were removed from the analysis (Tables 2 and 3).

**Table 1:** Presentation of the study populations with health outcomes, work tasks and ergonomic and psychosocial conditions

	Forklift operators				Office workers				
Variable	(*n* = 150)	Mean	SD^a^	%^b^	(*n* = 44)	Mean	SD	%	*P* Value
Demographics									
Men	110			73	24			55	0.04
Women	40			27	18			41	0.04
Age (range:21–66)		40.4	9.60			37.3	9.42		0.70
Length of employment		9.8	4.41			7.3	5.25		< 0.01
Health and lifestyle									
Has experienced neck pain	74			49	13			30	0.02
Neck pain during the previous 7 days	42			28	4			9	0.48
Intensity of neck pain		5.04	2.47			5.0	2.00		0.90
Sick leave due to neck pain	26			17	0			0	< 0.01
Shoulder pain	95			63	17			39	< 0.01
Shoulder pain during the previous 7 days	53			35	4			9	0.03
Intensity of shoulder pain		5.28	2.52			4.36	2.29		0.14
Perceived good health	121			81	39			89	0.08
Ever smoked	84			56	30			68	0.15
Work tasks									
Has previously worked as forklift operator^c^	150			100	18			41	< 0.01
Low-lifting order picker	123			82	14			32	< 0.01
Counterbalanced tilting mast	129			86	15			34	< 0.01
Reach decker	100			67	11			25	< 0.01
Never sits at work	56			37	7			16	0.02
Sits at work part of the day	42			28	8			18	0.08
Sits at work 100%	52			35	29			66	< 0.01
Works in drafts or cold	69			46	5			11	< 0.01
Floor bumps when operating forklift	69			46	5			11	< 0.01
Neck extension	108			72	9			20	< 0.01
Neck lateral flexion	104			69	8			18	< 0.01
Neck rotation	124			83	12			27	< 0.01
Monotonous arm/ hand movements	119			79	19			43	< 0.01
Heavy lifting arms/ hands	112			75	13			30	< 0.01
Work involving hands above shoulder level	100			67	7			16	< 0.01
Psychosocial factors									
Satisfied with work		7.15	1.94			8.1	1.40		0.04
Co-worker support	135			90	43			98	< 0.01
Freedom of work	105			70	39			89	< 0.01
Stress due to high work pace		5.2	2.82			5.4	2.81		0.64
Physically strenuous work		13.6	2.63			8.8	2.34		< 0.01
Work ability now compared with personal best		7.1	1.98			8.6	1.43		< 0.01

Chi squared cross-tabulations (categorical variables) and t-tests (continuous variables) for comparisons between the groups

^a^Standard deviation

^b^Missing values not taken into consideration when calculating percentages

^c^Office workers may operate forklifts on rare occasions

**Table 2:** Unadjusted odds ratios^a^ for ever having had neck pain, shoulder pain or combined after various exposures during current employment

	Neck pain *n* = 71		Shoulder pain *n* = 95		Combined *n* = 106	
Risk factors	OR	95% CI	OR	95% CI	OR	95% CI
Age years						
18–32	Reference level		Reference level		Reference level	
33–37	0.88	0.37–2.07	0.90	0.39–2.11	0.69	0.29–1.65
38–46	1.45	0.62–3.38	0.95	0.40–2.23	1.08	0.44–2.63
47–66	1.13	0.47–2.68	1.29	0.55–3.00	1.11	0.45–2.75
Female gender	1.00	0.52–1.94	1.07	0.56–2.05	1.13	0.57–2.24
Forklift operator	5.19	1.48–18.2	3.89	1.44–10.5	3.26	1.29–8.29
Extension	3.77	1.92–7.40	2.56	1.39–4.74	3.22	1.70–6.10
Rotation	3.94	1.82–8.54	3.22	1.64–6.32	3.09	1.56–6.09
Lateral flexion	3.30	1.71–6.36	2.65	1.43–4.91	2.86	1.51–5.40
Monotonous work hands arms	4.00	1.79–8.95	3.16	1.57–6.37	3.52	1.74–7.12
Heavy physical work	2.28	1.12–4.61	3.29	1.67–6.46	2.99	1.52–5.88
Work involving hands above shoulder level	2.91	1.53–5.55	3.33	1.79–6.20	3.78	1.97–7.24
Stress due to high work pace	2.62	1.29–5.30	1.56	0.82–2.97	2.03	1.05–3.92
Has previously smoked	1.62	0.70–3.74	2.88	1.16–7.18	2.36	0.90–6.23

^a^Odds ratios from univariate logistic regression analysis using perceived neck pain, shoulder pain and combined as dependent variable and potential risk factors as independent

**Table 3:** Incidence rate ratios for neck pain by years of exposure adjusted for age and gender, psychosocial aspects and hand/shoulder work

	A = age and gendSer		B = A+ Psychosocial aspects		C = A+ Hand/shoulder work	
Neck postures and exposure time	IRR^a^	95% CI^b^	IRR	95% CI	IRR	95% CI
Extension						
Non exposed	Reference level		Reference level		Reference level	
1–2 years	**3.09**	**1.08–8.86**	1.23	0.97–8.85	2.09	0.70–6.28
3–4 years	**5.34**	**2.42–11.78**	**4.63**	**2.00–10.72**	**3.56**	**1.52–8.36**
5–9 years	**2.01**	**1.00–4.03**	1.89	0.91–3.92	1.38	0.63–2.99
10- years	1.04	0.48–2.20	1.05	0.48–2.31	0.71	0.32–1.60
Rotation						
Non exposed	Reference level		Reference level		Reference level	
1–2 years	**7.10**	**2.63–19.14**	**5.86**	**2.08–16.50**	**6.24**	**1.97–19.79**
3–4 years	**5.70**	**2.47–13.27**	**5.00**	**2.09–11.99**	**4.76**	**1.67–13.65**
5–9 years	1.70	0.78–3.74	1.49	0.67–3.31	1.58	0.62–4.04
10- years	1.24	0.53–2.91	1.13	0.47–2.71	1.11	0.39–3.14
Lateral flexion						
Non exposed	Reference level		Reference level		Reference level	
1–2 years	**5.60**	**2.23–14.09**	**4.51**	**1.67–12.13**	**4.35**	**1.68–11.29**
3–4 years	**3.76**	**1.80–7.80**	**3.54**	**1.67–7.49**	**3.06**	**1.40–6.69**
5–9 years	1.55	0.79–3.05	1.37	0.68–2.77	1.30	0.64–2.66
10- years	0.90	0.41–1.94	0.84	0.37–1.89	0.72	0.31–1.64

Bold numbers are those statistically significant at 95% confidence level

Poisson multivariate regression model. Person years were calculated from years of employment and years of exposure up to the debut of perceived neck pain

^a^Incidence rate ratio

^b^95% Confidence interval. Column B; Psychosocial factors included: mental stress, lack of co-worker support and freedom to perform your work. Column C; Hand/shoulder work includes monotonous work with arms/hands, heavy lifting and work involving hands above shoulder level

Taking person years into consideration, the risk from exposure was calculated in a multivariate Poisson regression model. The total length of employment was calculated for each individual, either ending in 2014 or up to the first year of perceived neck pain. The exposure time was calculated from the total amount of time spent with the neck in non-neutral postures. Minimum exposure time is one calendar year. The numbers of cases in the exposed and unexposed groups were calculated. The two groups were analyzed in a regression model in order to obtain incidence rate ratios (IRR) (Table 3). All analyses were adjusted for age and gender. Poisson and the binominal regression analyses were performed with the software STATA 12 (Statistical Software StataCorp LP, US). A probability value of 0.05 (5%) was considered statistically significant. Possible confounding factors were those variables shown in Table 2 to be risk factors for neck pain. Collinearity was tested in a nonparametric Spearman’s rank correlation test for every item adjusted for in the multiple regression models. To avoid interference between correlating predictors, the multiple regression analysis was carried out in three steps adjusted for different categories of work-related exposure.

## Results

The characteristics of the study populations, consisting of forklift operators and office workers, are presented in Table 1. Their occupational titles are those held at the time of the questionnaire. The study presents data concerning the demographics, illness, lifestyle, exposure and psychosocial factors of forklift operators and office workers. A total of 194 employees (of whom two did not state their gender) completed the questionnaire and participated in the analysis, i.e. 45% (see “study population” above). The average age of forklift operators was 40.4 years (SD 9.6, range: 21–66) and of office workers 37.3 years (SD 9.4, range 20–63). Average length of employment was 9.8 years (SD 4.4) for forklift operators and 7.3 years (SD 5.2) for office workers. Seventy-four forklift operators (49%) and 13 (30%) office workers replied that they had at some time in adult life experienced neck pain. Intensity of neck pain on a scale from 1 to 10 was similar in both groups (mean 5). Experience of neck pain during the seven days before answering the questionnaire was reported by 28% of the forklift operators and 9% of the office workers (n.s.). Ever having been on sick leave for neck pain was significantly more common among forklift operators (17%) than among office workers (0%) *p* < 0.001. For shoulder pain, see Table 1. Many office workers started as forklift operators before later switching to office work, which explains why 41% of the office workers reported that they had at some time operated a forklift truck. Working with the head in unnatural positions (extended, in lateral flexion and/or rotated) was significantly more common among forklift operators than among office workers. Thirty-five percent of the forklift operators and 66% of the office workers reported sitting for 100% of the working day. Thirty-seven percent of the forklift operators and 16% of the office workers reported that they did not sit at all. Twenty-eight percent of forklift operators said that they sat for part of the day compared with 18% of office workers (Table 1).

Work satisfaction, on a 10 degree scale, was higher for office workers (8.1) than for forklift operators (7.15) (*p* = 0.04). Good co-worker support was reported by 90% of the forklift operators as compared with 98% of the office workers (*p* < 0.001). Eighty-nine percent of office workers reported that they were free to decide how to perform their work, as compared with 70% of forklift operators 70% (p < 0.001). There were no reported differences in perceived stressed from a high work pace. Forklift operators had a significantly higher physical workload than office workers, 13.6 versus 8.8 *p* < 0.01 on a 20-item scale. Current work ability compared to peak ability was significantly lower among forklift operators than in office workers: 7.1 versus 8.6 p < 0.01 on a 10-item scale.

Odds ratios (OR) for potential risk factors are presented in Table 2. Ever having worked as a forklift operator is associated with an increased risk of having neck pain (OR = 5.19; 95 CI 1.48–18.2. Holding the head in unnatural positions also resulted in an increased risk of neck pain; extended (OR = 3.77; 95% CI 1.92–7.4), rotated (OR = 3.9; 95% 1.8–8.5 CI) or laterally flexed (OR =3.3; 95% 1.7–6.4 CI). Monotonous hand and arm work, heavy manual work and manual work tasks above shoulder-level as well as mental stress were associated with neck pain. There was no significant association between age or gender and neck pain (Table 2). Similar results were obtained for shoulder pain (Table 2).

In an incident rate ratio (IRR) analysis of neck pain we looked at length of exposure to unnatural neck positions (Table 3), with person years up to the first year of neck pain or the end of the study period for the exposed group and the corresponding time for the unexposed subjects. The results reveal that 1–2 years of neck extension at work leads to a higher risk of neck pain than the corresponding length of employment for non-exposed workers (IRR = 3.09; 95% CI 1.08–8.9). Three to four years of neck extension further increased the risk of neck pain (IRR = 5.34; 95% CI 2.4–11.8). Of all the types of exposure in this model – extension, rotation and lateral flexion of the neck – the first four years of exposure are most strongly associated with increased risk of neck pain, when age and gender have been taken into account (Table 3, column A).When the psychosocial factors of mental stress, lack of co-worker support and freedom to perform work are taken into account in the Poisson regression, the IRR for neck pain from unnatural neck positions were somewhat weakened (Table 3, column B). When ergonomically strenuous hand/arm work are included in the Poisson regression, the IRR for neck pain from extension, 3–4 years, still presents an increased risk (IRR = 3.56; 95% CI 1.52–8.36) (for more details, see Table 3, column C). Exposure to rotation as a forklift operator for at least one year resulted in a significantly increased risk of neck pain; for rotation 1–2 years (IRR = 5.86; 95% CI 2.08–16.5).

## Discussion

Even working as a forklift operator with one’s head in unnatural positions for a couple of years resulted in a significantly increased risk of developing neck pain compared to those working with their heads in natural positions. In this study, the work title ‘forklift operator’ was used as a proxy for holding one’s head in unnatural positions, while the title ‘office worker’ was used as a proxy for more neutral head positions. The use of work titles makes it possible to translate ergonomic neck exposures into occupational titles rather than angles of neck position. This also helps us to understand and improve the ergonomic situation of forklift operators. An increased risk of neck pain was observed after 1–2 years of exposure; this then gradually reduced after 5–9 years of exposure. The reduced incident risk ratios by increasing number of years can be explained by the fact that forklift operators who developed neck pain were eventually obliged to leave the workplace. Since this is a retrospective cohort study rather than a prospective one, we are not able to include those workers who left. We can, however, make the assumption that those who left were more likely to be suffering from neck or other localized pain. This would involve a bias towards the null. But despite this phenomenon, the risks from operating forklifts remain.

The workplace where the study was performed is a major international company which offers many opportunities for career development. This explains why so many office workers have previously worked as forklift operators. It would have been interesting to evaluate the harmful effects of the three types of forklift truck (low-lifting order picker, reach decker and counterbalanced tilting mast). However, the operators switched between vehicles in order to get a more varied working day, which means that we cannot distinguish the effects of the individual types of vehicle.

Other studies have analyzed the association between neck positions and neck pain. Van den Heuvel et al. [15] looked at neck extension and found an association with neck and shoulder symptoms (OR =2.4 significant) in a group of office workers. The odds ratio for neck extension among forklift operators in our study is 3.7 (Table 2). The result seems reasonable, even with higher risk estimates up to 5.34 (Column A, Table 3), since forklift operators are exposed to more strenuous neck positions than office workers.

Van den Heuvel et al. [15] also studied the effects of neck rotation on neck pain risk among office workers. Their group found a crude OR of 2.6, significant, at rotation over 45 degrees for 14–45% of working time, while Marcus et al. [16] found only a slightly elevated risk among computer users (HR 1.17, N.S.) and Ariens et al. [5] reported an RR of 1.4 (N.S.) in their mixed occupation cohort. Again, the neck pain risk we found from rotation in a group of forklift operators (OR 3.9, Table 2) seems reasonable given that forklift operators are more exposed to unnatural head positions than the two other cohorts studied. For lateral flexion we found an OR of 3.3 (Table 2). To the best of our knowledge, the only previous study to have evaluated the association between neck/shoulder pain and lateral neck flexion, by Marcus et al. [16], found a non-significant association. However, they chose a minimal tilt angle for their study (> 3 degrees). According to our workplace observations of the forklift operators, exposure to lateral flexion, rotation and extension was more pronounced in our study than in a study by Marcus et al. [16].

We have chosen office workers as the reference group. A review of neck pain among office workers by Paksaichol et al. [17] was based on seven prospective cohort studies. They found only female gender and previous neck pain to be strong risk factors for the onset of new episodes of neck pain. Our reference group of office workers had a higher proportion of women (41%) than the exposed group of forklift operators (27%). This is a possible negative confounder which may reduce the effects of the ergonomic exposure among the forklift operators. We have therefore adjusted for gender in Table 3. We have also adjusted for age since it is a common risk factor for pain. These adjustments could explain the higher risks observed in Table 3 than in Table 2, where there is no adjustment for age and gender.

Psychological factors associated with neck pain are also commonly discussed in the literature. In one review, McLean et al. [18] report high job demands and poor social work support among office workers as strong risk factors for neck pain. In a review of office workers by Deokhoon et al. [19], they found that low satisfaction with the workplace environment, low work task variation, keyboard position and self-perceived muscular tension, were risk factors for developing neck pain. In our study we included some of these psychosocial work exposures, but they did not greatly affect the risk of neck pain from unnatural neck positions (Table 3, Column B). Since previous studies have identified risk factors for neck pain to be work positions which involve holding the hands above shoulder level [2, 8]; heavy physical work [2, 4] and monotonous hand/arm work [4, 20], we included these ergonomic exposures in our Poisson regression. Here, the risks from the unnatural neck positions remained, albeit somewhat weaker (Table 3, Column C).

In our study we examined the effect of three unnatural positions: extension, lateral flexion and rotation. We did not focus on exposure to neck flexion, since our study group of forklift operators does not assume that position very frequently. Previous studies give considerable support to the contention that the effects of exposure to neck flexion are harmful [4, 5, 21, 22]. When comparing prevalence of ever having had neck pain in our material (49% of forklift operators and 30% of office workers, Table 1) with studies of workers in the general population, the annual prevalence of neck pain varies from 27% in Norway to 33% in the UK and 47% in Quebec, Canada [23]. The annual prevalence of neck pain varies between occupations. A recent Swedish study shows that neck pain is common among workers in public dentistry, with an annual prevalence of 60–67% [24]. Cote et al. [23] showed in their review that neck pain ranged from 17% among dentists to 72% among dental hygienists. For office workers, the one-year prevalence of neck pain varied from 17.7% among Norwegian administrative workers to 63% among Swedish secretaries. For blue-collar workers, the annual prevalence of neck pain among Swedish crane operators is 74% [23]. Working with the cervical spine in flexion for long periods of time may increase the risk of neck pain. In a study by Marcus et al. [16], people exposed to a head position which involves tilting the head at an angle greater than 3° were 50% more likely to develop neck/shoulder pain than people who were not exposed to this kind of head position. In conclusion, Cote P et al. [23] found that neck pain is an important cause of disability. Likewise they found that physical and psychosocial exposures at the workplace contribute to an increased risk of cervical pain. Another review by McLean et al. [18] evaluated the link between occupational exposures and neck pain. They found an association between neck pain and psycho-social factors apart from age and earlier neck injury. Most occupational risk factors were only of limited evidence. The one-week prevalence in our material was 28% among the forklift operators and 9% among the office workers. In Sweden generally, the one-week prevalence of neck pain varies from 7% of office workers to 53% of female plant workers in the laminate industry [23]. When comparing different prevalence rates found in the literature it is important to bear in mind that the questionnaires used in the various studies will have differed. Comparisons within studies will therefore be more accurate than those between studies.

### Methodology

The strengths of this study are the long follow-up time (with a mean exposure time of 9.8 years for the forklift operators) and the fairly homogenous observed exposure situation. The exposure took place at two large warehouses belonging to one large company. A weakness of the study is the overall response rate of 45%. Our recruiting method was to invite all workers to participate and answer a questionnaire about experienced health and work environment. We invited every worker to participate independently of health status. We did not specifically approach those with neck pain. The study was supported both by the union and the human resource manager, who informed and encouraged all workers at their staff meetings to participate in the study. In the invitation the workers were guaranteed that their questionnaire answers would not be made available to anyone other than the research team. Three reminders were given. The response rate was 40% for the forklift operators and 60% for the office workers. The difference in response rates can probably be explained by the fact that it is likely to be easier for office workers than for forklift operators to answer a questionnaire. However, one can also imagine that those forklift operators who do not have neck pain might be less motivated to answer the questionnaire than their colleagues who do have symptoms. This would give falsely high risk estimates for unnatural neck positions. To correct for possible skewedness we have added the number of person years in the group of forklift operators who did not have neck pain by 50% as for simulating the same answer percentage in the forklift operators as in the office workers. By adding person years only in the non-symptomatic group we have constructed a worst-case biased scenario. This simulation gives an IRR for extension of the neck IRR = 2.7, 95% CI 1.1–6.4. Thus, the risks associated with unnatural neck positions remain. Another explanation for the low response frequency is the high percentage of immigrants with a poor knowledge of Swedish among the forklift operators. However, this would not bias the final results, since there is no reason to assume that the immigrant workers would have a higher or lower proportion of neck pain than their native Swedish colleagues.

We have conducted a retrospective cohort study with one exposed group, forklift operators, which we compare with a less exposed group, office workers. However, a certain degree of misclassification occurred since some office workers said that they had been exposed to unnatural neck positions. This is shown in Table 1, where we present a cross-sectional perspective. Thus, a non-differential misclassification has occurred, which would lower the prevalence rate ratios presented in Table 2. In Table 3, where we used person years as a measure of exposure to unnatural neck positions instead of occupational title, the misclassification is minimized.

Information about exposure was obtained by means of a questionnaire. We did not measure the angles of head rotation, extension or lateral flexion. We did, however, spend several days observing the subjects at work. We systematically recorded that the forklift operators held their heads in unnatural positions for most of their working hours, unlike the office workers, who spent most of their working time with natural head positions. In this study we have focused on the outcome variable neck pain. Our definition of neck pain is the answers of the respondents to the question “Do you have or have you had pain, ache or discomfort in your neck?” combined with a question about year of start and end of pain. This is a broad question which is intended to include workers with different degrees of pain intensity. We used the answers to this question to calculate the prevalence of ever having had neck/ or shoulder pain as a forklift operator or office worker in Table 1. Table 2 presents the odds ratios for prevalence for these two occupational groups.

Respondents give a more precise description of their pain in a question which asks about the intensity of pain in the previous seven days, on a scale from 0 = no pain up to 10 = pain as intense as pain can be. Mean pain intensity among forklift operators and office workers was similar, about 5 (Table 1). We have also reported risk indicators for neck pain and shoulder pain in Table 2. Because we asked about year of start and end of neck pain, we could calculate years of exposure before neck pain onset, the results of which are presented in Table 3. To avoid interference between correlating predictors, the multiple regression analysis was performed in three steps adjusted for different categories of work-related exposure.

There is an association between neck and shoulder pain because they share many of the same work-related risk factors. These include awkward postures, unnatural, static positions and mental stress due to high work pace. The forklift operators use both neck and shoulder muscles in their work. How muscle activation patterns and cervical range of motion are related to neck and shoulder pain could be studied further for this group of workers. No clinical or radiological examinations were performed so diagnosis using ICD-10 was not carried out. The diagnoses of the respondents were not elucidated. In the Swedish health care system, neck pain can be given a variety of classifications such as cervicalgia ICD code M 54.2; cervico-brachial syndrome ICD code M 53.1 (if pain radiates into the arms); and cervicocranial syndrome M53.0 (if the neck pain radiates up into the head). In some cases, a specific diagnosis can identified, such as herniated discs in the cervical spine ICD code M 50.1; spondylosis M47, or spinal stenosis M48. For a more precise analysis of the correlation between unnatural neck positions and specific diagnoses it would have been necessary to perform a thorough medical and radiological examination with Magnetic Resonance Imaging (MRI). Although this was not possible in our study, we nevertheless consider it valuable to try to establish the association between unnatural head positions and neck pain. Neck pain is a highly distressing condition. In our clinical experience, all advances in our understanding of how the risk of neck pain can be reduced are important, even if precise anatomical diagnoses are not available.

The sample size of our study is not very large. However, since the risk of neck pain from the exposure is high and the proportion of exposed subjects is also high, the number of subjects in this study is sufficient for us to be able to draw conclusions. Thus, the statistical significance tests resulted in the lower 95% confidence intervals exceeding the null for a large part of the associations. When working with a binary outcome, sample size is important for achieving sufficient statistical power. When one is addressing specific exposures, their proportion must also be taken into consideration: the exposed group should be at least 30% of the total number of participants in the analysis [25]. These are requirements are fulfilled in our study.

### Practical implications

The present study is, to the best of our knowledge, the first ever published about the association between forklift operating and neck pain. The results are in line with other studies of unnatural neck positions at work. There are 150,000 forklift operators in Sweden according to the manufacturing industry and Statistics Sweden. In Europe there are some six million forklift operators and globally about 20 million. The numbers are calculated by multiplying the numbers of forklift trucks sold every year by eleven (i.e. the lifetime of a forklift truck) multiplied again by two (the number of operators using one truck during a work shift) [26]. According to our results, 49% of forklift operators suffer from periodic neck pain. Furthermore, 28% reported neck pain during the previous week. Neck pain is obviously a major problem for forklift operators; it impacts their personal life as well as society.

The designers of forklift vehicles are aware of the problem and are working on solutions. We hope that this study will encourage them to prioritize the development of better-designed and more ergonomic vehicles.

Even 1–2 years of forklift driving with the head in an unnatural position increases the risk of neck pain. The condition is handicapping and makes some kinds of work impossible to perform. Efforts should therefore be made to improve the working conditions of forklift operators; this includes physical and organizational factors as well as improving operator ergonomics. Since a forklift machine is used for about eleven years, it is worthwhile looking at how the machines already in use can be improved while we wait for new, ergonomically-superior forklift trucks to be available.

## Conclusions

This is the first published study showing that forklift operators have an increased risk of neck pain. Even 1–2 years of forklift driving increases the risk of neck pain. Efforts should therefore be made to improve the working conditions of forklift operators. The results are of significance for improving work schedules and adjustment of work tasks for about 150,000 workers in Sweden and six million in Europe and, should also be of interest for those designing the vehicles.

## Abbreviations

CI: Confidence interval
ICD: International Statistical Classification of Disease and Related Health Problems
IRR: Incident rate ratio
MRI: Magnetic resonance imaging
N.S: Non significant
OR: Odds ratio
SD: Standard deviation
